# Resources and Personal Adjustment for Career Transitions Among Adolescents: A Latent Profile Analysis

**DOI:** 10.1002/jad.12507

**Published:** 2025-04-20

**Authors:** Anna Parola, Jenny Marcionetti

**Affiliations:** ^1^ Department of Humanities University of Naples Federico II Naples Italy; ^2^ University of Applied Sciences and Arts of Southern Switzerland Locarno Switzerland

**Keywords:** adolescence, career adaptability, career transition, latent profile analysis, personal adjustment, resilience, vision about future

## Abstract

**Introduction:**

Career transitions are considered to be the most challenging tasks in adolescence. Personal resources are important factors in coping with the difficulties encountered during transitions and help individuals to adjust more smoothly to these transitions. Using a person‐centered approach, this study aims to identify typologies of personal resources in adolescents, that is career adaptability, hope, optimism and resilience and their association with personal adjustment.

**Methods:**

Six hundred and twenty six Italian adolescents (*M* = 17.18; SD = 0.52) participated in the study. First, a latent profile analysis was conducted using personal resources. Then, a MANOVA was conducted to capture the association of resource profiles with life satisfaction and anxiety/depression.

**Results:**

The results of the profile analysis revealed four profiles of personal resources. Pessimists (*n* = 123), Unbalanced (*n* = 63), Career Maladjusted (*n* = 187), and Career Adjusted (*n* = 253). The profile with high resources (Career Adjusted) show a higher personal adjustment, while profiles with low resources show lower personal adjustment. In particular, the Unbalanced profile, characterized by the lowest level of hope and resilience, shows the lowest personal adjustment with medium‐high anxiety/depression and low life satisfaction.

**Conclusions:**

The results suggest that career adaptability resources alone may not be sufficient to promote good personal adaptation and therefore the readiness to cope with career transitions in adolescence, but that these should be accompanied, in particular, by good levels of hope and resilience.

## 1 Introduction

From a life‐span developmental psychology perspective, the social and economic context is of fundamental importance in determining the nature, timing, and outcomes of adolescent transitions (Baltes et al. 1980; Bynner and Parsons 2002; Lerner 1982; Lerner et al. 1996). Transitions necessarily refer to the various developmental turning points at which the individual is called upon to make a transition by responding to specific developmental tasks, imposing a new life direction through the restructuring of daily routines and relevant transformations in personal identity (Rumbaut 2005). Among these, one aspect that has been widely studied in the literature is the lengthening of the transition from school to work in recent decades and the increasing complexity that young people encounter in this transition (Bynner 2012; Bynner and Parsons 2002). Indeed, this transition does not follow a linear path and is not triggered by a single moment of decision but is a complex and fragmented process influenced by young people's negotiation skills (Goodwin and O'Connor 2005).

Individuals' experience of school‐to‐work transition has changed considerably in recent years as a result of the current changing contexts. The work patterns typical of 20th century societies, characterized by more secure employment and more stable organizations, which provided a more secure basis for building life paths and imagining a future, has given way to the new arrangements of the 21st century, characterized instead by uncertainty and instability in the labor market and fluid organizations (Kalleberg et al. 2000). Postmodernity has had a significant impact not only on the lengthening of the school‐to‐work transition (Bynner 2012), but also on the number of career transitions that individuals have to make throughout their lives. As stated by Kulcsár et al. (2020, 2), “they may involve choosing an occupation and the educational training involved, then a job and then whether to stay at a job or switch to another one, what formal and informal advanced training to take, and so on…”. In this perspective, which focuses more on lifelong career development and the lifelong process of shaping one's own life, adolescents and young people need to acquire knowledge and competences related to the process of making choices due to different choices throughout the life span. Postmodernity has also influenced the now more active role of young people in constructing their career paths (Duarte 2004; Savickas 2007; Savickas 2012). Specifically, Nota et al. (2020) and Guichard (2018) emphasize the need to stimulate adolescents to give meaning to their individual existence, taking into account global challenges and the impact of their own active lives on others. Awareness of current challenges is the first step to eliciting new types of future identity forms, previously hidden or unknown. The work of awareness and meaning making is possible owing to personal resources that help individuals to overcome barriers that might limit their range of choices. Currently, young people have to make career choices while facing three types of macro‐challenges that have an impact at a social, political and economic level. The first is technological evolution and digitalization. The world is facing the fourth industrial revolution (also known as Industry 4.0). It builds on the third and is characterized by a fusion of technologies that blur the boundaries between the physical, digital, and biological spheres (Schwab 2016). Industry 4.0 is characterized by advances in artificial intelligence, robotics, the Internet of Things, nanotechnology, biotechnology, quantum computing, and block chain (Schwab 2016). Among the challenges posed by the Fourth Industrial Revolution, Schwab (2016) highlights in particular that of inequality, particularly because of its potential to disrupt labor markets. The second challenge is related to the economic recession and the resulting labor market problems. In Europe, young people aged 15–34 are the most vulnerable in the labor market, with the highest risk of unemployment and underemployment. In 2023, an average of 11.2 per cent were identified as NEET (not engaged in education, employment or training) in the EU (Eurostat 2024). The highly‐contextualized nature of the phenomenon is most evident in Mediterranean countries, with the highest rates recorded in Greece and Italy, where 16% or more of all young people were NEET. Problems in the labor market make it difficult to build a sustainable career, to find decent work and to build a decent life (Blustein et al. 2019). Related to this is the mismatch between the education system and the world of work. WorldSkills & Organization for Economic Co‐operation and Development OECD (2019) conducted a survey of adolescents and young people in 19 countries to find out whether they are confident about finding a job and whether they are receiving sufficient educational support to prepare them for the future. Around 56% of young people know what they want to do in the future and 50% say they are optimistic about finding a job in the future. However, they do not feel supported by the education system: 44% fear that their skills or knowledge will not be in demand in the labour market in the future and 71% of young people would like more help/support with career choices at school. Skills mismatch, as an imbalance between the supply and demand of skills, leads to unemployment (Asai et al. 2020). Finally, all environmental threats. This category can be defined as all the environmental and contextual challenges that have a profound impact on young adults' vision of the future in today's society. These include climate change and natural disasters such as earthquakes, as well as pandemics and wars. The psychological literature traces how climate change (Hickman et al. 2021), fear and worry of war (Regnoli et al. 2024; Regnoli et al. 2025), and pandemics (Parola 2020) are associated with the perception of “no future.” Indeed, these highlighted challenges make it difficult not only to live in the present, but also to aspire and imagine a possible future (Di Maggio et al. 2021; Guichard 2018).

But how does postmodernity shape young people's career transitions in practice? In the scenario described above, adolescents and young adults are trying to deal with these various important challenges that threaten the future of humanity (Cohen‐Scali et al. 2018; Pouyaud and Guichard 2017), trying to prevent them from becoming obstacles in their transition to the world of work. The threats addressed, which can be defined as the main “concerns” of the young people in transition, have in common that they undermine the labour market and make it difficult for individuals to have a desired future. Savickas highlights several consequences of the postmodernity: “The new job market in an unsettled economy calls for viewing career not as a lifetime commitment to one employer but as a recurrent selling of services and skills to a series of employers who need projects completed” (2012, 13). Adolescents and young people in transition should expect to have at least ten jobs in their lifetime (Wehmeyer et al. 2019).

The current situation has highlighted the need to re‐adapt students' ability to cope with the threats and ongoing challenges. Addressing these needs, which involve different aspects of life, requires a holistic and complex approach. Career Construction Theory (CCT; Savickas 2013) emphasizes the need to support individuals in co‐constructing and planning their personal and professional careers, and Life Design Practices, based on CCT, is a theoretical and practical approach to career guidance that aims to promote people's adaptability in contexts of extreme uncertainty, nonlinearity and crisis (Savickas et al. 2009).

### 1.1 Theoretical Framework: The Career Construction Theory

In light of the changes described above, it seems necessary to define what new skills are needed for the school‐to‐work transition and to adopt a life‐course perspective to study in depth the antecedents of career development as key elements of adjustment and well‐being (McMahon and Watson 2008; Porfeli et al. 2008; Schultheiss 2008). As mentioned above, the linear development of the worker's life has been abandoned, and individuals are expected to constantly make new choices about their careers, to constantly learn new skills, to change many jobs over the course of their lives, and to be frequently involved in rethinking their life course.

Career construction theory (CCT; Savickas 2013) has adapted to understand this societal shift. Career development is complex and subject to multiple influences, both external and internal and this mutability provides opportunities for people to become more active in constructing their own futures (Nota et al. 2020). Savickas (2013) describes three developmental roles of the self in career construction: the actor, who learns social norms in the family environment; the agent, who extends to the community and school by adapting to change; and the author, who unifies goals and intentions in a personal narrative. A central aspect of CCT is the role of personal resources. Given the complexity and instability of the environment, it is difficult to operate within it, so it is up to individuals to use their resources to adapt to this fluidity. A successful career transition may depend on the individual's ability to cope with the various contextual conditions associated with the transition and to make full use of their psychological resources.

The Life Design paradigm (Savickas et al. 2009) emphasizes the relevance of complex and nonlinear career trajectories and provides theoretical and methodological guidance to help individuals respond to educational and work contexts that tend to change suddenly and can be fluid and unstable. In this framework, life is conceived as a design, in a continuous work of planning, flexibility and openness to change. Therefore, the demand for proactivity on the part of the individuals is continuous throughout their lives and also in the construction of their careers (Nota and Rossier 2015). The Life Design framework implements the previous theories in a lifelong, holistic, contextual and preventive model. Lifelong because the paradigm considers trajectories throughout an individual's life, holistic because it considers the whole individual, and contextual because it considers the context in which transitions occur (Savickas et al. 2009). In addition, the Life Design paradigm is conceived as a preventive tool, acting before the person is faced with transitions (Savickas et al. 2009). Therefore, the promotion of positive resources becomes a central element of the life design approach. On the one hand, it plays a preventive role and, on the other hand, it allows the individual to grow in a positive way. This is particularly true and important to consider for youth development (Wehmeyer et al. 2019).

### 1.2 Personal Resources for Career Transitions: Career Adaptability, Hope, Optimism and Resilience

According to the CCT, optimal career outcomes are achieved with psychosocial resources (Savickas 2013). Career outcomes, defined as “adaptation outcomes,” are directly and indirectly predicted by adaptation resources such as career adaptability and adaptation readiness, which include resources such as hope and optimism (Rudolph et al. 2017). Individuals in transition should be willing to change in the face of the change they face (adaptive readiness), use personal resources to change (adaptability resources), take action to keep up with the change (adapting responses), and thus demonstrate adaptation.

#### 1.2.1 Career Adaptability

The core resource of the CCT is career adaptability defined as the ability to respond to the complexity and constant change of the socioeconomic environment. Adaptability is a multi‐component resource consisting of four characteristics: concern, control, curiosity and confidence (Savickas 2013). Concern indicates the ability to orient oneself towards the future. Control is the ability to manage one's career choices. Curiosity is the ability to manage information about career opportunities. Finally, confidence is related to the sense of self‐efficacy in constructing one's career.

Adaptability implies the ability to adjust to unexpected needs arising from changes in the labor market and/or working conditions (Savickas and Porfeli 2012). This means that individuals should be able to respond to change by using their adaptability. It is a process through which people dynamically construct their working lives, managing change, and taking into account the context in which they live (Karaevli and Tim Hall 2006; Savickas 2005). In a literature review on career adaptability, Johnston (2018) traces four possible correlates: personality, particularly conscientiousness (Rossier et al. 2012; Teixeira et al. 2012), self factors, such as anxiety (Pouyaud et al. 2012) and self‐esteem (Rusu et al. 2015; van Vianen et al. 2012), positive adjustment and coping (Tolentino et al. 2014; van Vianen et al. 2012), and factors related to positive career development, such as career aspirations, frequency of career exploration behaviors, and career decisions (Urbanaviciute et al. 2014).

#### 1.2.2 Hope

Hope can be defined as an individual's attitude towards changes in the contemporary labor market. It consists of two crucial aspects, namely agency thinking, which refers to the determination to initiate and sustain the effort required to achieve goals and aspirations, and path thinking, which is required to achieve a goal (Snyder et al. 1991). It is a “guidance‐drive resource” in that it helps individuals to plan goals, mobilize other resources, and enact behaviors to achieve goals. In career development, the use of hope is crucial for individuals because it enables them to maintain a positive vision of their future and to stay focused on goals by pursuing them vigorously. Several studies suggest a positive effect of hope on career development processes and outcomes (Santilli et al. 2014; Santilli et al. 2017; Maree 2013). Furthermore, hope is particularly important for the achievement of career goals among disadvantaged groups (Diemer and Blustein 2007). Hope for the future should be fostered to counteract the negative outlooks that young people may passively inherit from their environment (Fusco et al. 2021).

#### 1.2.3 Optimism

Optimism also appears to be a useful resource in unpredictable contexts. Optimism is understood as a general expectation of positive future outcomes (Bryant and Cvengros 2004; Ginevra et al. 2017). From this perspective, hope and optimism in adolescents can help them to constructively engage in educational and career planning (Ginevra et al. 2017). Optimism allows individuals to manage the direction of their lives and subjective experiences within the boundaries of active engagement (Schneider 2001). Several studies have shown that optimism about the future is a positive resource that works to increase an individual's employability (Ginevra et al. 2017; Zammitti et al. 2023).

#### 1.2.4 Resilience

Finally, resilience can also be seen as the ability to cope with the uncertainties of contemporary society (Sapienza and Masten 2011). Traditionally, resilience has been defined as a process by which individuals demonstrate positive adaptation in the face of adversity or trauma (Luthar and Cicchetti 2000). Many studies have suggested that resilience can be both proactive and reactive (Bonanno 2004; Luthans et al. 2006). According to Luthans et al. (2006), resilience is an element of one's psychological capital. Specifically, it involves the development of coping strategies to overcome career obstacles (Bimrose and Hearne 2012; Cardoso and Moreira 2009). According to London (1983), resilience is the key ability to adapt to changing and very adverse circumstances that could discourage individuals or put them at risk of being trapped in vulnerable trajectories. In addition, Coetzee (2008) suggests that resilience acts as a control, keeping the career drivers in balance so that people do not go overdo (or burn out) in constructing their careers.

### 1.3 Social Resources to Career Transitions: The Role of Parents

The literature identifies different sources of perceived social support for career development at different developmental stages. During adolescence, parents are the most important source of support (Keller and Whiston 2008). Indeed, parental figures serve as a source of information and emotional support during transitions from one educational system to another. In particular, Parola and Marcionetti (2022) have shown that parental support is most needed during the high school period. At the end of high school, especially in some contexts such as Italy, young people have to make the most difficult choice: whether to enroll in university or to enter the world of work. Although this is not the first career choice, which is made at the end of secondary school, it is the most difficult one for adolescents to make, because it involves more indecision. Parental support turns out to be crucial in this choice, more so than in other stages of life (Parola and Marcionetti 2022). Several studies have shown that parental support increases self‐efficacy in making career choices (Kush and Cochran 1993; Lent et al. 2003; Wolfe and Betz 2004) and promotes career exploration (Kracke 1997) and competences (Commodari et al. 2022; Commodari et al. 2024). Therefore, parents can be an important resource for adolescents, by providing instrumental and emotional support (Marcionetti and Rossier 2016).

### 1.4 Relationship Between Resources and Personal Adjustment

As mentioned above, career adaptability, hope, optimism, and resilience are resources theoretically intended to act as a shield against the difficulties that adolescents and young people may encounter in making career transitions in the context in which these transitions take place. Together, they allow individuals to protect themselves against barriers and obstacles of the current labor market. In fact, the literature shows that these resources are connected and influence each other. Associations have emerged in adolescence between career adaptability and hope (e.g. Buyukgoze‐Kavas 2016; Santilli et al. 2017), optimism (e.g. Tolentino et al. 2014) and resilience (e.g. Drosos and Antoniou 2023; Ginevra et al. 2017; Santilli et al. 2020). Studies therefore show that career adaptability resources are positively correlated with constructs related to goal pursuit and those that lead to positive adjustment and coping (Johnston 2018).

Specifically, career adaptabilities and hope have common aspects (Korkmaz 2023): one of the components of hope is pathway, which refers to strategies to achieve the goal (Snyder 2002) and career adapt‐abilities also refer to the strategies used by the individual in managing critical tasks, transitions, and traumas (Savickas 2013). Hope, as a resource, helps people to believe that they will be able to take concrete steps to achieve future goals. Individuals with lower levels of hope tend to avoid tasks that are necessary to achieve their goals (Snyder 2002). In the same way, several studies suggests that optimism might be an antecedent of career adaptability (Tolentino et al. 2014). Adolescents and young people who report higher levels of optimism are better able to cope with work‐ or career‐related challenges and changes (Buyukgoze‐Kavas 2016; Duffy 2010). Finally, according to Rossier et al. (2017), in the LD paradigm resilience is most closely related to career adaptability and is a fundamental personal resource that helps people shape their professional lives. Together, career adaptability and career resilience promote adaptive functioning and successful lives (Maree 2017).

These resources are protective factors for the individual well‐being. In particular, career adaptability promotes the development of a sense of power (Hirschi 2009) and the experience of life satisfaction (Hirschi 2009; Marcionetti and Rossier 2021). Moreover, career adaptability, which encourages individuals to reflexively imagine and construct a life with viable and multiple roles, contributes to ensuring well‐being and adaptive functioning in adolescence (Xu et al. 2020). Similarly, hope for a positive future and resilience contribute to well‐being in adolescence (Ginevra et al. 2017; Santilli et al. 2020; Xu et al. 2020) and reduce mental health problems such as anxiety and depression (Xu et al. 2020). In particular, hope has been widely studied as a predictor of optimal functioning and shown to be associated with better adjustment in students (Wong and Cheung 2024).

In addition to personal resources, contextual resources can be critical in determining how transitions are managed, particularly parental figures. Parents can be a valuable resource for adolescents in transition when they provide instrumental and emotional support (Marcionetti and Rossier 2016; Parola et al. 2023) and promote their life satisfaction (Parola and Marcionetti 2022).

### 1.5 The Current Study

This study aims to identify which personal resource profiles emerge in adolescents and to analyse their combination with some indicators of personal adaptation, namely life satisfaction and anxiety/depression. Previous studies with a person‐centred approach have focused on profiling specific resources, such as career adaptability profiles (Bouckenooghe et al. 2022; Hirschi and Valero 2015). To our knowledge, no previous studies have attempted to consider how some key resources in educational and career transitions defined by CCT, such as career adaptability, hope, optimism and resilience, interact to shape different profiles. To this end, the person‐centred approach was considered the best methodological approach, also in light of the increasing individualisation of the career process (Hirschi and Valero 2015; Spurk et al. 2020). This approach allows to capture profiles that may differ quantitatively (level of the dimensions) and qualitatively (shape of the profile) (Hirschi and Valero 2015; Wang and Hanges 2011). While the variable‐centered approach assumes that resources and personal adjustment manifest itself in the same way across a population, the person‐centered approach leaves open the possibility that resources may manifest in different combinations across individuals. Finally, to test the utility of the profiles in predicting relevant outcomes, we examined whether individuals belonging to different profiles of resources also differ in their personal adjustments. Identifying adolescent profiles with different combinations of resources and their personal adjustment could improve understanding of developing dynamics and provide information for career guidance interventions.

In summary, we used latent profile analysis to identify different resource profiles in adolescents. Parental support was considered a possible antecedent in the construction of the profile. The latent profiles that emerged were finally compared in light of their association with indicators of personal adjustment, i.e. with anxious/depressive symptoms and life satisfaction. We expected this:

− At least one profile of high career adaptability, hope, optimism, and resilience.
− At least one profile of low career adaptability, hope, optimism, and resilience.
− Adolescents with profiles characterized by high career adaptability, hope, optimism, and resilience would report less anxious/depressive symptoms and higher life satisfaction.

## 2 Methods

### 2.1 Participants and Data Collection

The sample consisted of 626 Italian adolescents (*M* = 17.18; SD = 0.52), 322 males and 304 females, in their final year of high school. Overall, most of the participants' parents had completed secondary education (87%) and at least one parent was employed (77%). 94% of the adolescents reported being of Italian nationality. Participants were recruited through university‐school cooperation networks involving five different high schools in southern Italy. Before data collection, permission to administer anonymous self‐report questionnaires was obtained from the school principals. Parental consent was obtained for participants under the 18 years of age. Participants were informed of the purpose of the study, assured of the confidentiality of their responses, and given the opportunity to withdraw from the study at any time. Data collection took place in the first trimester of 2023. The study was approved by the Ethics Committee of the University of Naples Federico II to ensure the protection of participants.

### 2.2 Measures

#### 2.2.1 Demographic Background

Students were asked to provide demographic information about their gender, age, nationality, and socioeconomic level (SES).

#### 2.2.2 Personal Resources

To assess the career adaptability, the Career‐Adaptability Scale (CAAS; Savickas and Porfeli 2012) validated in Italian by Soresi et al. (2012) was used. Participants are asked to rate on a 5‐point Likert scale ranging from 1 (not strong) to 5 (strongest). The scale assesses the four dimensions of career adaptability: concern (6 items), control (6 items), curiosity (6 items) and confidence (6 items). High scores on the total score indicate greater career adaptability. Examples of items are: “Counting on myself,” “Looking for opportunities to grow as a person.” The Cronbach alpha in this study was 0.89.

To assess hope and optimism, the Vision About Future (VAF; Ginevra et al. 2017) scale was used. The Hope scale consists of 7 items; the Optimism scale consists of 6 items. Participants are asked to answer on a 5‐point Likert scale ranging from 1 (It doesn't describe me at all) to 5 (It describes me very well). Examples of items are: “I feel that I will get quite well” (hope scale), “Usually, I am full of enthusiasm and optimism about my future” (optimism scale). The Cronbach alpha in this study was 0.85 for hope and 0.78 for optimism.

To assess resilience, the Design My Future (DMF; Di Maggio et al. 2016) scale was used. The resilience scale consists of 11 items. Participants are asked to answer on a 5‐point Likert scale ranging from 1 (not at all) to 5 (very well). Example of item is “I consider myself able to tackle everything that may happen.” The Cronbach alpha in this study was 0.91.

#### 2.2.3 Social Resources

To assess parental support, the Parental Career‐Related Behaviors Questionnaire (PCB, Parola et al. 2023) was used. The support scale consists of 5 items. Participants are asked to rate them on a 4‐point Likert scale ranging from 1 (does not apply) to 4 (fully applies). Example of items is “My parents give advice on the choice of careers available.” The Cronbach alpha in this study was 0.85.

#### 2.2.4 Personal Adjustment

To assess the satisfaction with one's life, the Satisfaction with Life Scale (SWLS; Diener et al. 1985; Di Fabio and Palazzeschi 2012) was used. The scale consists of 5 items. Participants are asked to use a 7‐point Likert scale ranging from 1 (strongly disagree) to 7 (strongly agree). Example of item is: “The conditions of my life are excellent.” The Cronbach alpha in this study was 0.83.

To assess anxiety and depression, the Youth Self Report (YSR; Achenbach and Rescorla 2001) scale was used. The anxiety/depression scale consists of 13 items. Participants are asked to answer on a 3‐point scale ranging from 1 (not true) to 3 (very true or often true). Example of item is: “There is very little that I enjoy.” High scores indicate high levels of reported anxiety and depressive problems. Cronbach alpha in this study was 0.73.

### 2.3 Data Analyses

First, means, standard deviations, correlation analysis between the study variables and tests of normality assumption (skewness and kurtosis) were performed. The missing completely at random test (MCAR; Little 1988) was used to test the assumption of random missing values. The results indicated that the percentage of missing data did not exceed 5% and Little's test was not significant (*p* = 0.138), supporting the assumption that the missing values were missing completely at random. Missing data were treated in the analysis using full information maximum likelihood (FIML) (Little and Rubin 2020).

Second, latent profile analysis (LPA) was used to identify resource profiles. A robust maximum likelihood estimator (MLR) was selected. The analysis used the standardized variables due to the diversity of response modes in the measures used. Career adaptability, hope, optimism, and resilience were selected in the model. A stepwise approach was used to select the optimal number of profiles, starting with two profiles and gradually increasing the number of latent classes until convergence problems arose or the goodness of fit criteria suggested that additional classes were unlikely to yield valid results (Nylund et al. 2007). The LPA is a model testing process and each model is compared with the previous model(s) to make a decision about the number of latent profiles in the data (Christie and Masyn 2008). The decision regarding model retention in the LPA was made using the Akaike Information Criterion (AIC; Akaike 1987), the Bayesian Information Criterion (BIC; Schwarz 1978), and the Sample‐size Adjusted BIC (SABIC; Sclove 1987). Lower values of AIC, BIC, and SABIC indicate the best fitting models (Feldman et al. 2009). In addition, further tests and indicators were used to evaluate the number and reliability of the profiles. The Lo‐Mendell‐Rubin adjusted likelihood ratio test with *p* > 0.05 (LRT; Lo et al. 2021) and the bootstrapped likelihood ratio test with *p* > 0.05 (BLRT; McLachlan 2000) were used to assess the parsimony of classes. Specifically, the LRT and the BLRT tests compare a model with k profiles to a model with a k‐1 profile, and a significant *p* value indicates that a model with k profiles has a better fit than a model with a k‐1 profile. The entropy statistic was used to assess the model‐based classification accuracy. Higher entropy values indicate improved enumeration accuracy and, therefore, a clear profile separation (Nagin 2005). Entropy values between 0.60 and 0.80 were considered good (Jung and Wickrama 2008). Average posterior probabilities were examined to assess the accuracy of each classification into its most likely class, with higher probabilities close to 1 indicating greater confidence in class membership. Finally, the number of subjects per latent profile was considered, taking into account the recommendation that profiles containing less than 5% of the sample may be spurious (Masyn 2013).

Third, multinomial logistic regression was performed using the R3STEP command to examine whether gender, SES and parental support predicted profile membership. For SES, a composite score was created by averaging the scores for maternal and paternal education and gross annual household income (as reported by the adolescents in the questionnaire). For inclusion in the analysis, the composite scores were converted into low, medium and high SES.

Finally, after identifying the typologies resources, a multivariate analysis of variance (MANOVA) was used to determine between‐profile differences in adolescents' life satisfaction and anxiety/depressive symptoms (namely personal adjustment). This procedure, following the LPA, makes it possible to examine the relationship between profile membership and outcome variables that were not used to determine profile membership. In this study, each outcome variable served as a dependent variable in the MANOVA, and profile membership served as an independent variable that captures whether individuals assigned to the identified resource profile report different levels of life satisfaction and anxiety/depression symptoms.

Initial analyses and MANOVA were performed with the SPSS software (version 29), whereas LPA, and multinomial logistic regression were conducted using the Mplus software (version 8).

## 3 Results

Table 1 presents the descriptive analysis (means and standard deviations) of this study sample. The skewness and kurtosis values indicate the normality of the data distribution. Correlations are shown in Table 2. All the resources are positively correlated with each other and with life satisfaction and negatively correlated with anxiety/depression.

**Table 1:** Descriptive statistics for study variables.

Measure	*M*	SD	Sk	K
1. Career adapt.	3.662	0.610	−0.103	0.059
2. Hope	4.511	0.567	−1.985	0.376
3. Optimism	3.420	0.571	−0.722	0.364
4. Resilience	3.013	0.804	−0.013	‐0.65
5. Parental support	3.198	0.613	−0.681	0.432
6. Anx./Dep.	5.896	5.783	0.926	0.031
7. Life satisf.	4.797	1.241	−0.457	‐0.082

Abbreviations: K, Kurtosis; M, mean, SD, Standard Deviation; S*k*, Skewness.

**Table 2:** Correlations for study variables.

Variable	1	2	3	4	5	6	7
1. Career adaptability	—						
2. Hope	0.114**	—					
3. Optimism	0.338***	0.302***	—				
4. Resilience	0.156**	0.193***	0.148***	—			
5. Parental support	0.334***	0.190***	0.871***	0.142***	—		
6. Anxiety/depression	−0.160***	−0.231***	−0.126**	−0.593***	−0.111*	—	
7. Life satisfaction	0.347***	0.173***	0.327***	0.213***	0.326***	−0.160***	—

** *p* < 0.01

*** *p* < 0.001.

The LPA was performed using a stepwise procedure, and the four‐profile solution appeared to be the best solution, as adding another class (five‐profile) did not improve the model fit (Table 3). The AIC and SABIC were lower in the four‐profile solution than in the five‐profile solution. In addition, the entropy values were lower in the progression from the fourth to the fifth profile (0.910 for the four‐profile solution and 0.872 for the five‐profile solution). Entropy indicates how well each LPA model divides the data into profiles (Celeux and Soromenho 1996) and being high for the four‐profile solution indicated confidence in supporting this profiling. Furthermore, the nonsignificant LRT test for the five‐profile solution suggested that the more parsimonious model is the better fitting and representative model (Ferguson et al. 2020). Looking at the partitioning of subjects within profiles, the five‐profile solution presented a profile characterized by 17 individuals, violating the recommendation of at least 5% of the sample for each profile. According to Ferguson et al. (2020), when a small number of participants from the sample are represented in a profile, it is difficult to be confident that the profile represents a distinct typology that can be generalized to other samples.

**Table 3:** LPA fit statistics.

Fit statistics	2‐Class	3‐Class	4‐Class	5‐Class
AIC	6850.890	6615.682	6573.384	6517.508
BIC	6798.530	6695.590	6675.489	6641.810
SABIC	6867.328	6638.443	6602.467	6552.914
Entropy	0.985	0.889	0.910	0.872
LRT *p* value	< 0.001	< 0.001	< 0.001	0.140
BLRT *p* value	< 0.001	< 0.001	< 0.001	< 0.001

**Figure 1:**
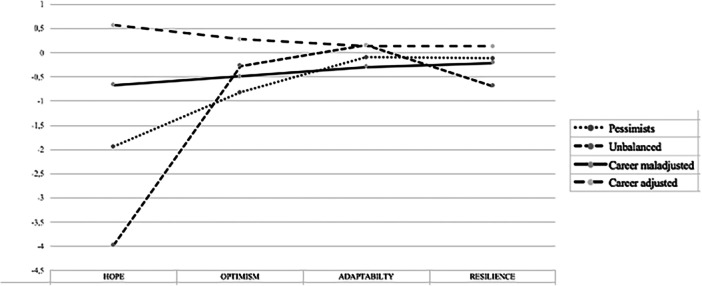
Profiles of personal resources.

The four‐profile solution showed the best fit to the data (AIC = 6573.384; BIC = 6675.489; SABIC = 6602.467), a good entropy value (0.910) and a significant *p* value of LRT and BLRT. The posterior probabilities were close to 1 for each profile, ranging from 0.914 to 0.974. The four‐profile solution is shown in Figure 1. Profile 1 (*n* = 123) was labeled “Pessimists.” In this profile, individuals have lower levels of career adaptability, resilience and hope, and the lowest levels of optimism. Profile 2 (*n* = 63) was labeled “Unbalanced.” In this profile, individuals show moderate levels of career adaptability, while endorsing lower levels of optimism and the lowest levels of hope and resilience. Profile 3 (*n* = 187) was labeled “Career maladjusted.” In this profile, individuals have lower levels of hope, optimism and resilience and the lowest level of career adaptability. Profile 4 (*n* = 253) was labeled “Career adjusted.” In this profile, the individuals show the highest levels of career adaptability, hope, optimism and resilience.

The results of the multinomial logistic regression results with Profile 4 (Career adjusted) as the reference group showed no significant differences for SES and gender between the profiles. Instead, parental support significantly predicted belonging to Profile 1 (b = −0.978, SE = 0.438, *p* = 0.026, OR = 0.376, 95%CI: 0.159−0.887), that is to the “Pessimists” profile, and Profile 3 (b = −0.742, SE = 0.135, *p* < 0.001, OR = 0.476, 95%CI: 0.365–0.620), that is, to the “Career maladjusted” profile, indicating a higher likelihood of being in these profile for those perceiving low parental support.

We then examined whether adolescents with different resources profiles differed in their personal adjustment. Table 4 summarizes the results of the MANOVA. The results showed that personal adjustment differed between the profiles for both life satisfaction [F (3, 617) = 10.218, *p* < 0.001, η^2^ = 0.047] and anxiety/depression [F (3, 617) = 8.456, *p* < 0.001, η^2^ = 0.039].

**Table 4:** Results of MANOVA comparing clusters.

Measure	Pessimists		Unbalanced		Career maladjusted		Career adjusted		*F*(3, 617)	η^2^
	*M*	SD	*M*	SD	*M*	SD	*M*	SD		
Life satisfaction	4.44	1.15	4.19	1.85	4.80	1.24	4.99	1.21	10.218***	0.047
Anxiety/Depression	6.32	5.58	8.18	5.56	11.65	7.05	5.32	5.65	8.456***	0.039

Abbreviations: *M*, mean; SD, Standard Deviation.

*** *p* < 0.001

## 4 Discussions

Adolescents face many threats and challenges related to their future careers and are called upon to make the most important career choices at the end of high school. To make these choices, individuals need to use all the resources at their disposal to cope with the current transitional environment. According to the CCT (Savickas 2013; Rossier et al. 2017), an adolescent copes better when there are adequate levels of career adaptability, a positive view of the future (hope and optimism) and resilience. Although studies have used a variable‐centered approach to explore the relationship between these resources, this approach does not take into account that within an adolescent population there may be several distinct subpopulations with different resource profiles.

Indeed, the results of the profile analysis revealed four profiles of personal resources. Two profiles (Profile 3 and Profile 4) are mirrored, showing either high resources and therefore high career adaptability, high hope, high optimism and high resilience (Career adjusted), or low resources and therefore low career adaptability, low hope, low optimism and low resilience (Career maladjusted). The high resources profile represents individuals who also show higher personal adjustment (i.e. high life satisfaction and low anxiety/depression levels). In this case, the resources are adaptive for the individual and could lead to positive career outcomes. In particular, career adaptability could act as a meta‐skill (Tolentino et al. 2014) that activates and brings with it a positive outlook on the future and resilience. In contrast, the low‐resources profile represents maladjusted individuals who lack the resources to face the challenges and changes imposed by the environment and thus show particularly high anxiety/depression levels. Thus, in line with our hypothesis, at least one high resource profile and at least one low resource profile were found.

Additionally, Profile 1 (Pessimists) is characterized by low levels of all resources, and particularly of hope and optimism. The Pessimist profile appears to be one in which the vision of the future seems to be particularly negative, with negative effects on the life satisfaction and anxiety/depression levels of those having this resources profile. Finally, Profile 2 is characterized by the presence (although not at a high level) of career adaptability which, however, is not followed by high resourcefulness, but on the contrary by low optimism and the lowest levels of hope and resilience. In contrast to the other profile, this one, defined as ‘Unbalanced’, does not show a balance (in quantitative terms) of resources. Individuals with this profile of resources are those showing the lowest levels of life satisfaction and the highest levels of anxiety/depression.

In terms of possible antecedents, the results revealed a significant association between parental support and a higher likelihood of belonging to the Pessimists and Career maladjusted profiles. Hence, in line with the literature which found that low perceived support is associated with low career adaptability (Parola and Marcionetti 2022), hope (Zeng et al. 2023), optimism (Sumer et al. 2009) and resilience (Zeng et al. 2023), this study further confirms that a lack of parental support is associated with a lack of positive resources in adolescence. Concerning the transition from study to work, parental support is essential to provide guidance on how to formulate specific career goals and make educational and career choices that support these goals (Hargrove et al. 2002; Liang et al. 2020). Conversely, problematic parenting behaviour exacerbates career indecision and further complicates an already complicated career decision‐making process (Parola et al. 2023). To explain the results of this study, it is perhaps useful to consider the recent study by Zhou et al. (2024) that suggests that adequate development of career adaptability is associated with specific career support from parents, but also with a positive parent‐child relationship.

Our study found no differences related to gender, which is consistent with other studies that do not support differences in career resources between men and women (Hirschi 2009). With regard to hope and optimism, the literature is controversial, to the point of confirming Boman and Yates (2001) assertion that it is almost impossible to accurately describe gender similarities and differences in adolescence in terms of optimism and pessimism. We didn't find an effect of the SES either. Some studies showed that it affects opportunities in terms of resources and even career options available to adolescents (Juntunen et al. 2020). It is likely that in this study the effect of the SES is completely mediated by perceived parental support. SES can in fact influence parental support (Lee 2018), which in turn can influence belonging to a certain resource profile, as in the case of Profile 1 (Pessimists).

Profile 3 (Unbalanced) is the second highest in terms of anxiety/depression and the lowest in terms of life satisfaction. This finding is unexpected as adaptability is seen as a resource that brings adjustment and well‐being (Savickas et al. 2009). This finding has two implications: on the one hand, career adaptability alone, if not accompanied by a positive outlook on the future and resilience, fails in its function as a protective factor; on the other hand, it invites us to break down the single dimension into its components (concern, control, curiosity and confidence). Although previous studies suggest that the four dimensions are strongly correlated and follow the same constellation (high, medium and low) (Bouckenooghe et al. 2022; Hirschi and Valero 2015), our data show higher means in the control dimension (M = 3.69) than in the other dimensions. Control refers to taking responsibility for one's own career development. Probably adolescents with this profile feel the responsibility for their career development, but less an orientation towards exploration of the future self and career opportunities (curiosity) and confidence in problem solving. In this case, there is likely to be a sense of individual responsibility for the future, without necessarily being positive and hopeful about it. Indeed, the levels of hope in this profile are very low compared to those found in the other profiles and invite a deeper reflection on the role of hope in building the future. Hope opens up the future, “enables us to break out of closed time as a prison” (Han 2024, p.5). Again, ‘to hope means to put one's trust in reality, to believe in it so that it carries with it a future.’ (Han 2024, p.6). Hope, as a personal resource, can give meaning and direction to young people, even those belonging to disadvantaged groups (Diemer and Blustein 2007) or living in a challenging environment (Fusco et al. 2021), fostering their career development processes and outcomes (Santilli et al. 2014; Santilli et al. 2017; Maree 2013). For this reason, when it is profoundly absent, as in this profile, it can lead to low levels of life satisfaction and high levels of internalizing symptoms such as anxiety and depression, even when career adaptability is present. Additionally, the difference in scores between hope (low) and optimism confirms the evidence often found in the literature of a difference between the two constructs. Optimism is a disposition of the individual, while hope is an openness to a possible and unknown future. Hope, even in terms of career intervention, is open to narrative, which is why its absence makes it difficult to imagine future life and career plans with the adolescent. Finally, this result could also be explained (and read) by the specificity of the context, that is, Southern Italy. Although career studies suggest that career flexibility is an element to be promoted to help young people achieve positive outcomes in their life course, studies in Southern Italy suggest a positive association between career flexibility in adolescence and anxiety and depression (Fusco et al. 2021). These studies suggest that rather than pushing young people to passively adapt to the characteristics of the labor market in contexts where a stagnant economy is clearly present (Fusco et al. 2022), career practitioners should seek to reinforce hope in the future and help young people living in such a negative environment to shape a future that will enable them to achieve desirable developmental outcomes.

Overall, these findings have important implications for career intervention. First, in line with the Life Design paradigm, they highlight the importance of working on resources, and particularly on hope, to achieve personal adjustment. In this sense, hope is a promising avenue for intervention to promote a smoother career transition and facilitate students' adjustment (Wong and Cheung 2024). Second, they indicate adolescents perceiving a lack of parental support among those more at risk to lack personal resources and to be less satisfied with their lives. As highlighted by La Grange and Maree (2022), it is important to work on these resources for those who have experienced parental neglect (e.g. lack of involvement in children's career choices). In this case, life design interventions should focus on turning pain into hope to improve meaning making and enhance innate resilience and adaptability. Third, they are useful to understand the relationship between adaptability and other resources in the individual. Indeed, career adaptability resources alone may not be sufficient if one does not also has a positive vision of the future and feel resilient in dealing with situations that may arise in the current context. Therefore, despite the centrality of this construct in CCT, if the objective is to promote a good level of well‐being to be able to face the challenges inherent in career transitions, it seems important that the career counsellor's work focus first on fostering the development of such resources in the adolescent (Parola and Marcionetti 2020).

This study also has its limitations. First, it uses a convenience sample of adolescents from Southern Italy. This limitation requires caution in the interpretation of the results and calls for the planning of future studies that may instead allow for the generalizability of the results. Second, the study uses a cross‐sectional design. Future studies should adopt a longitudinal design to determine the temporal patterns of profile membership during career transitions (whether the resource profiles change over time or whether belonging to a certain resource profile can change over time) and outcomes (e.g., adaptive school‐to‐work transition or reduced decision‐making difficulty in specific career choices). Finally, our results indicate that it would be worthwhile to further investigate the association between the perceived quality of the relationship between parents and adolescents and belonging to the resource profiles that emerged.

In conclusion, this study suggests the need to promote developmental assets in children and adolescents to contribute to their personal adjustment. In the current challenging scenario, the main aim of career practitioners should be to rekindle hope and devise strategies to turn hope into action (Maree 2022).

## Ethics Statement

The study was approved by the Ethics Committee of Psychological Research, Department of Humanistic Studies, University of Naples Federico II, approved on 13.05.2019 (Prot no. 20/2019).

## Conflicts of Interest

The authors declare no conflicts of interest.

## Data Availability

The data that support the findings of this study are available from the corresponding author (Anna Parola) upon reasonable request.

## References

[jad12507-bib-0001] Achenbach, T. M. , and L. A. Rescorla . 2001. Manual for the ASEBA School‐Age Forms & Profiles. University of Vermont, Research Center for Children, Youth, and Families.

[jad12507-bib-0002] Akaike, H. 1987. “Factor Analysis and AIC.” Psychometrika 52: 317–332.

[jad12507-bib-0003] Asai, K. , T. Breda , A. Rain , L. Romanello , and M. Sangnier . 2020. “Education, Skills and Skill Mismatch. A Review and Some New Evidence Based on the PIAAC Survey.” In Rapport IPP n°, 26. Institut des Politiques Publiques.

[jad12507-bib-0004] Baltes, P. B. , H. W. Reese , and L. P. Lipsitt . 1980. “Life‐Span Developmental Psychology.” Annual Review of Psychology 31, no. 1: 65–110. 10.1146/annurev.ps.31.020180.000433 7362217

[jad12507-bib-0005] Bimrose, J. , and L. Hearne . 2012. “Resilience and Career Adaptability: Qualitative Studies of Adult Career Counseling.” Journal of Vocational Behavior 81, no. 3: 338–344.

[jad12507-bib-0006] Blustein, D. L. , M. E. Kenny , A. Di Fabio , and J. Guichard . 2019. “Expanding the Impact of the Psychology of Working: Engaging Psychology in the Struggle for Decent Work and Human Rights.” Journal of Career Assessment 27, no. 1: 3–28.

[jad12507-bib-0007] Boman, P. , and G. C. R. Yates . 2001. “Optimism, Hostility, and Adjustment in the First Year of High School.” British Journal of Educational Psychology 71, no. 3: 401–411. 11593947 10.1348/000709901158587

[jad12507-bib-0008] Bonanno, G. A. 2004. “Loss, Trauma, and Human Resilience: Have We Underestimated the Human Capacity to Thrive After Extremely Aversive Events?” American Psychologist 59, no. 1: 20–28. 14736317 10.1037/0003-066X.59.1.20

[jad12507-bib-0009] Bouckenooghe, D. , A. Kanar , and U. C. Klehe . 2022. “A Latent Transition Analysis Examining the Nature of and Movement Between Career Adaptability Profiles.” Journal of Vocational Behavior 136: 103728.

[jad12507-bib-0010] Bryant, F. B. , and J. A. Cvengros . 2004. “Distinguishing Hope and Optimism: Two Sides of a Coin, or Two Separate Coins?” Journal of Social and Clinical Psychology 23, no. 2: 273–302.

[jad12507-bib-0011] Buyukgoze‐Kavas, A. 2016. “Predicting Career Adaptability From Positive Psychological Traits.” The Career Development Quarterly 64, no. 2: 114–125.

[jad12507-bib-0012] Bynner, J. 2012. “Policy Reflections Guided by Longitudinal Study, Youth Training, Social Exclusion, and More Recently NEET.” British Journal of Educational Studies 60, no. 1: 39–52.

[jad12507-bib-0013] Bynner, J. , and S. Parsons . 2002. “Social Exclusion and the Transition From School to Work: The Case of Young People Not in Education, Employment or Training.” Journal of Vocational Behavior 60: 289–309.

[jad12507-bib-0014] Cardoso, P. , and J. M. Moreira . 2009. “Self‐Efficacy Beliefs and the Relation Between Career Planning and Perception of Barriers.” International Journal for Educational and Vocational Guidance 9: 177–188.

[jad12507-bib-0015] Celeux, G. , and G. Soromenho . 1996. “An Entropy Criterion for Assessing the Number of Clusters in a Mixture Model.” Journal of Classification 13: 195–212.

[jad12507-bib-0016] Christie, C. A. , and K. E. Masyn . 2008. “Latent Profiles of Evaluators' Self‐Reported Practices.” Canadian Journal of Program Evaluation 23: 225–254.

[jad12507-bib-0017] Coetzee, M. 2008. “Psychological Career Resources of Working Adults: A South African Survey.” SA Journal of Industrial Psychology 34, no. 2: 10–20.

[jad12507-bib-0018] Cohen‐Scali, V. , J. Rossier , and L. Nota . 2018. New Perspectives on Career Counseling and Guidance in Europe. Springer.

[jad12507-bib-0019] Commodari, E. , A. Consiglio , M. Cannata , and V. L. La Rosa . 2024. “Influence of Parental Mediation and Social Skills on Adolescents' Use of Online Video Games for Escapism: A Cross‐Sectional Study.” Journal of Research on Adolescence 34, no. 4: 1668–1678. 39438433 10.1111/jora.13034 PMC11606255

[jad12507-bib-0020] Commodari, E. , S. Platania , V. L. La Rosa , et al. 2022. “Psychological Well‐Being In Adolescence: Relationships Between Life Skills, Self‐Efficacy, and Metacognitive Skills.” Mediterranean Journal of Clinical Psychology 10, no. 1: 1–19.

[jad12507-bib-0021] Diemer, M. A. , and D. L. Blustein . 2007. “Vocational Hope and Vocational Identity: Urban Adolescents' Career Development.” Journal of Career Assessment 15, no. 1: 98–118.

[jad12507-bib-0022] Diener, E. , R. A. Emmons , R. J. Larsen , and S. Griffin . 1985. “The Satisfaction With Life Scale.” Journal of Personality Assessment 49: 71–75. 16367493 10.1207/s15327752jpa4901_13

[jad12507-bib-0023] Drosos, N. , and A. S. G. Antoniou . 2023. “Career Adaptability and Career Resilience in the Workplace.” In Resilience in Modern Day Organizations, 68–78. Routledge.

[jad12507-bib-0024] Duarte, M. E. 2004. “O Indivíduo E a Organização: Perspectivas De Desenvolvimento.” Psychologica (Extra‐Série): 549–557.

[jad12507-bib-0025] Duffy, R. D. 2010. “Sense of Control and Career Adaptability Among Undergraduate Students.” Journal of Career Assessment 18: 420–430.

[jad12507-bib-1006] Eurostat . 2024. Statistics on Young People Neither in Employment nor in Education or Training. https://ec.europa.eu/eurostat/statistics-explained/index.php?title=Statistics_on_young_people_neither_in_employment_nor_in_education_or_training.

[jad12507-bib-0027] Di Fabio, A. , and L. Palazzeschi. 2012. The Satisfaction With Life Scale (SWLS): Un contributo alla validazione italiana con lavoratori adulti. *Counseling: Giornale Italiano di Ricerca e Applicazioni*.

[jad12507-bib-0028] Feldman, B. J. , K. E. Masyn , and R. D. Conger . 2009. “New Approaches to Studying Problem Behaviors: A Comparison of Methods for Modeling Longitudinal, Categorical Adolescent Drinking Data.” Developmental Psychology 45: 652–676. 19413423 10.1037/a0014851 PMC2791967

[jad12507-bib-0029] Ferguson, S. L. , E. W. G. Moore , and D. M. Hull . 2020. “Finding Latent Groups in Observed Data: A Primer on Latent Profile Analysis in Mplus for Applied Researchers.” International Journal of Behavioral Development 44, no. 5: 458–468.

[jad12507-bib-0030] Fusco, L. , A. Parola , and L. S. Sica . 2021. “Life Design for Youth as a Creativity‐Based Intervention for Transforming a Challenging World.” Frontiers in Psychology 12: 662072. 34017294 10.3389/fpsyg.2021.662072 PMC8129198

[jad12507-bib-0031] Fusco, L. , L. S. Sica , A. Parola , and L. Aleni Sestito . 2022. “Vocational Identity Flexibility and Psychosocial Functioning in Italian High School Students.” International Journal of School & Educational Psychology 10, no. 1: 144–154.

[jad12507-bib-0032] Ginevra, M. C. , T. M. Sgaramella , L. Ferrari , L. Nota , S. Santilli , and S. Soresi . 2017. “Visions About Future: A New Scale Assessing Optimism, Pessimism, and Hope in Adolescents.” International Journal for Educational and Vocational Guidance 17: 187–210.

[jad12507-bib-0033] Goodwin, J. , and H. O'Connor . 2005. “Exploring Complex Transitions: Looking Back at the ‘Golden Age' of From School to Work.” Sociology 39, no. 2: 201–220.

[jad12507-bib-0034] La Grange, C. , and J. G. Maree . 2022. “Efficacy of Using Life Design‐Based Counselling for an Emerging Adult Who Had Suffered Parental Neglect.” South African Journal of Higher Education 36, no. 5: 179–201.

[jad12507-bib-0036] Guichard, J. 2018. “Life Design Interventions and the Issue of Work.” In Interventions in Career Design and Education: Transformation for Sustainable Development and Decent Work, edited by V. CohenScali , J. P. Pouyaud, M. Drabik‐Podgorna , et al., 15–28. Springer, Cham.

[jad12507-bib-0037] Han, B. C. 2024. The Spirit of Hope. John Wiley & Sons.

[jad12507-bib-0038] Hargrove, B. K. , M. G. Creagh , and B. L. Burgess . 2002. “Family Interaction Patterns as Predictors of Vocational Identity and Career Decision‐Making Self‐Efficacy.” Journal of Vocational Behavior 61, no. 2: 185–201.

[jad12507-bib-0040] Hickman, C. , E. Marks , P. Pihkala , et al. 2021. “Climate Anxiety in Children and Young People and Their Beliefs About Government Responses to Climate Change: A Global Survey.” Lancet Planetary Health 5, no. 12: e863–e873. 34895496 10.1016/S2542-5196(21)00278-3

[jad12507-bib-0041] Hirschi, A. 2009. “Career Adaptability Development in Adolescence: Multiple Predictors and Effect on Sense of Power and Life Satisfaction.” Journal of Vocational Behavior 74, no. 2: 145–155.

[jad12507-bib-0042] Hirschi, A. , and D. Valero . 2015. “Career Adaptability Profiles and Their Relationship to Adaptivity and Adapting.” Journal of Vocational Behavior 88: 220–229.

[jad12507-bib-0043] Johnston, C. S. 2018. “A Systematic Review of the Career Adaptability Literature and Future Outlook.” Journal of Career Assessment 26, no. 1: 3–30.

[jad12507-bib-0044] Jung, T. , and K. A. S. Wickrama . 2008. “An Introduction to Latent Class Growth Analysis and Growth Mixture Modeling.” Social and Personality Psychology Compass 2, no. 1: 302–317.

[jad12507-bib-0045] Juntunen, C. L. , S. R. Ali , and K. R. Pietrantonio . 2020. “Social Class and Poverty: A Renewed Focus in Career Development.” In Career Development and Counseling: Putting Theory and Research to Work, edited by S. D. Brown and R. W. Lent , 341–373. Wiley.

[jad12507-bib-0046] Kalleberg, A. L. , B. F. Reskin , and K. Hudson . 2000. “Bad Jobs in America: Standard and Nonstandard Employment Relations and Job Quality in the United States.” American Sociological Review 65, no. 2: 256–278.

[jad12507-bib-0047] Karaevli, A. , and D. T. Tim Hall . 2006. “How Career Variety Promotes the Adaptability of Managers: A Theoretical Model.” Journal of Vocational Behavior 69, no. 3: 359–373.

[jad12507-bib-0048] Keller, B. K. , and S. C. Whiston . 2008. “The Role of Parental Influences on Young Adolescents' Career Development.” Journal of Career Assessment 16, no. 2: 198–217.

[jad12507-bib-0049] Korkmaz, O. 2023. “Will Hope and Career Adapt‐Abilities Bring Students Closer to Their Career Goals? An Investigation Through the Career Construction Model of Adaptation.” Current Psychology 42, no. 3: 2243–2254.

[jad12507-bib-0050] Kracke, B. 1997. “Parental Behaviors and Adolescents' Career Exploration.” Career Development Quarterly 45, no. 4: 341–350.

[jad12507-bib-0051] Kulcsár, V. , A. Dobrean , and I. Gati . 2020. “Challenges and Difficulties in Career Decision Making: Their Causes, and Their Effects on the Process and the Decision.” Journal of Vocational Behavior 116: 103346.

[jad12507-bib-0052] Kush, K. , and L. Cochran . 1993. “Enhancing a Sense of Agency Through Career Planning.” Journal of Counseling Psychology 40, no. 4: 434–439.

[jad12507-bib-0053] Lee, I. H. 2018. “The Link Between Socioeconomic Status and Career Adaptability Among Korean Adolescents: The Mediating Role of Parental Career‐Related Support.” Career and Technical Education Research 43, no. 1: 57–75.

[jad12507-bib-0054] Lent, R. W. , S. D. Brown , L. Nota , and S. Soresi . 2003. “Testing Social Cognitive Interest and Choice Hypotheses Across Holland Types in Italian High School Students.” Journal of Vocational Behavior 62, no. 1: 101–118.

[jad12507-bib-0055] Lerner, R. M. 1982. “Children and Adolescents as Producers of Their Own Development.” Developmental Review 2, no. 4: 342–370.

[jad12507-bib-1005] Lerner, R. M. , J. V. Lerner , A. von Eye , et al. 1996. “Continuity and Discontinuity Across the Transition of Early Adolescence: A Developmental Contextual Perspective.” In Transitions Through Adolescence: Interpersonal Domains and Context, edited by J. A. Graber , J. Brooks‐Gunn , A. C. Petersen , 3–22. Lawrence Erlbaum.

[jad12507-bib-0056] Liang, Y. , N. Zhou , K. Dou , et al. 2020. “Career‐Related Parental Behaviors, Adolescents' Consideration of Future Consequences, and Career Adaptability: A Three‐Wave Longitudinal Study.” Journal of Counseling Psychology 67, no. 2: 208–221. 32105127 10.1037/cou0000413

[jad12507-bib-0057] Little, R. J. A. 1988. “A Test of Missing Completely at Random for Multivariate Data With Missing Values.” Journal of the American Statistical Association 83, no. 404: 1198–1202.

[jad12507-bib-0058] Little, R. J. A. , and D. B. Rubin . 2020. Statistical Analysis With Missing Data. John Wiley & Sons.

[jad12507-bib-1002] Lo, Y. , N. R. Mendell , and D. B. Rubin . 2001. “Testing the Number of Components in a Normal Mixture.” Biometrika 88: 767–778.

[jad12507-bib-0060] London, M. 1983. “Toward a Theory of Career Motivation.” Academy of Management Review 8, no. 4: 620–630.

[jad12507-bib-0061] Luthans, F. , J. B. Avey , B. J. Avolio , S. M. Norman , and G. M. Combs . 2006. “Psychological Capital Development: Toward a Micro‐Intervention.” Journal of Organizational Behavior 27, no. 3: 387–393.

[jad12507-bib-0062] Luthar, S. S. , and D. Cicchetti . 2000. “The Construct of Resilience: Implications for Interventions and Social Policies.” Development and Psychopathology 12, no. 4: 857–885. 11202047 10.1017/s0954579400004156 PMC1903337

[jad12507-bib-0063] Di Maggio, I. , M. C. Ginevra , L. Nota , and S. Soresi . 2016. “Development and Validation of an Instrument to Assess Future Orientation and Resilience in Adolescence.” Journal of Adolescence 51: 114–122. 27348551 10.1016/j.adolescence.2016.06.005

[jad12507-bib-0064] Di Maggio, I. , M. C. Ginevra , S. Santilli , L. Nota , and S. Soresi . 2021. “Life Design for an Inclusive and Sustainable Future.” In The Palgrave Handbook of Positive Education, edited by M. L. Kern and M. L. Wehmeyer , 251–270. Palgrave Macmillan.

[jad12507-bib-0065] Marcionetti, J. , and J. Rossier . 2016. “The Parental Career‐Related Behaviors (PCB) Questionnaire: Italian Validation.” Testing, Psychometrics, Methodology in Applied Psychology 23: 347–363.

[jad12507-bib-0066] Marcionetti, J. , and J. Rossier . 2021. “A Longitudinal Study of Relations Among Adolescents' Self‐Esteem, General Self‐Efficacy, Career Adaptability, and Life Satisfaction.” Journal of Career Development 48, no. 4: 475–490.

[jad12507-bib-0067] Maree, J. G. 2013. “Latest Developments in Career Counselling in South Africa: Towards a Positive Approach.” South African Journal of Psychology 43, no. 4: 409–421.

[jad12507-bib-0068] Maree, J. G. 2022. “Managing the Covid‐19 Pandemic in South African Schools: Turning Challenge Into Opportunity.” South African Journal of Psychology 52, no. 2: 249–261.

[jad12507-bib-0069] Maree, K. 2017. “The Psychology of Career Adaptability, Career Resilience, and Employability: A Broad Overview.” In Psychology of Career Adaptability, Employability and Resilience, edited by K. Maree , Springer.

[jad12507-bib-0070] Masyn, K. E. 2013. “Latent Class Analysis and Finite Mixture Modeling.” In The Oxford Handbook of Quantitative Methods, edited by T. Little , 551–611. Oxford University Press.

[jad12507-bib-0071] McLachlan, G. 2000. Finite Mixture Models. A Wiley‐Interscience Publication.

[jad12507-bib-0072] McMahon, M. L. , and M. B. Watson . 2008. “Systemic Influences on Career Development: Assisting Clients to Tell Their Career Stories.” Career Development Quarterly 56, no. 3: 280–288.

[jad12507-bib-1001] Nota, J. , and J. Rossier . 2015. “Handbook of Life Design.” In From Practice to Theory and From Theory to Practice. Hogrefe.

[jad12507-bib-0073] Nagin, D. 2005. Group‐Based Modeling of Development. Harvard University Press.

[jad12507-bib-0074] Nota, L. , S. Soresi , I. Di Maggio , S. Santilli , and M. C. Ginevra . 2020. Sustainable Development, Career Counselling and Career Education. Springer.

[jad12507-bib-0075] Nylund, K. L. , T. Asparouhov , and B. O. Muthén . 2007. “Deciding on the Number of Classes in Latent Class Analysis and Growth Mixture Modeling: A Monte Carlo Simulation Study.” Structural Equation Modeling: A Multidisciplinary Journal 14, no. 4: 535–569.

[jad12507-bib-0076] Parola, A. 2020. “Novel Coronavirus Outbreak and Career Development: A Narrative Approach into the Meaning for Italian University Graduates.” Frontiers in Psychology 11: 2255. 33192751 10.3389/fpsyg.2020.02255 PMC7642812

[jad12507-bib-0077] Parola, A. , L. Fusco , and J. Marcionetti . 2023. “The Parental Career‐Related Behaviors Questionnaire (PCB): Psychometric Properties in Adolescents and Young Adults in the Italian Context.” Current Psychology 42, no. 17: 14376–14386.

[jad12507-bib-1003] Parola, A. , and J. Marcionetti . 2020. “Career Orientation: A Qualitative Study of the Best Practices in the Swiss Context.” Mediterranean Journal of Clinical Psychology 8, no. 3: 1–26.

[jad12507-bib-0078] Parola, A. , and J. Marcionetti . 2022. “Career Decision‐Making Difficulties and Life Satisfaction: The Role of Career‐Related Parental Behaviors and Career Adaptability.” Journal of Career Development 49, no. 4: 831–845.

[jad12507-bib-0079] Porfeli, E. J. , P. J. Hartung , and F. W. Vondracek . 2008. “Children's Vocational Development: A Research Rationale.” Career Development Quarterly 57, no. 1: 25–37.

[jad12507-bib-0080] Pouyaud, J. , and J. Guichard . 2017. “A Twenty‐First Century Challenge: How to Lead an Active Life Whilst Contributing to Sustainable and Equitable Development.” In Career Guidance for Social Justice, 31–45. Routledge.

[jad12507-bib-0081] Pouyaud, J. , E. Vignoli , O. Dosnon , and N. Lallemand . 2012. “Career Adapt‐Abilities Scale‐France Form: Psychometric Properties and Relationships to Anxiety and Motivation.” Journal of Vocational Behavior 80, no. 3: 692–697.

[jad12507-bib-0082] Regnoli, G. M. , A. Parola , and B. De Rosa . 2025. “Development and Validation of the War Worry Scale (WWS) in a Sample of Italian Young Adults: An Instrument to Assess Worry About War in Non‐War‐Torn Environments.” European Journal of Investigation in Health, Psychology and Education 15, no. 2: 24. 39997088 10.3390/ejihpe15020024 PMC11853760

[jad12507-bib-0083] Regnoli, G. M. , G. Tiano , and B. De Rosa . 2024. “How Is the Fear of War Impacting Italian Young Adults' Mental Health? The Mediating Role of Future Anxiety and Intolerance of Uncertainty.” European Journal of Investigation in Health, Psychology and Education 14, no. 4: 838–855. 38667809 10.3390/ejihpe14040054 PMC11049055

[jad12507-bib-0085] Rossier, J. , M. C. Ginevra , G. Bollmann , and L. Nota . 2017. “The Importance of Career Adaptability, Career Resilience, and Employability in Designing a Successful Life.” Psychology of Career Adaptability, Employability and Resilience: 65–82.

[jad12507-bib-0086] Rossier, J. , G. Zecca , S. D. Stauffer , C. Maggiori , and J.‐P. Dauwalder . 2012. “Career Adapt‐ Abilities Scale in a French‐Speaking Swiss Sample: Psychometric Properties and Relationships to Personality and Work Engagement.” Journal of Vocational Behavior 80: 734–743.

[jad12507-bib-0087] Rudolph, C. W. , K. N. Lavigne , and H. Zacher . 2017. “Career Adaptability: A Meta‐Analysis of Relationships With Measures of Adaptivity, Adapting Responses, and Adaptation Results.” Journal of Vocational Behavior 98: 17–34.

[jad12507-bib-0088] Rumbaut, R. G. 2005. “Turning Points in the Transition to Adulthood: Determinants of Educational Attainment, Incarceration, and Early Childbearing Among Children of Immigrants.” Ethnic and Racial Studies 28, no. 6: 1041–1086.

[jad12507-bib-0089] Rusu, A. , C. Măirean , A. M. Hojbotă , L. R. Gherasim , and S. I. Gavriloaiei . 2015. “Relationships of Career Adaptabilities With Explicit and Implicit Self‐Concepts.” Journal of Vocational Behavior 89: 92–101.

[jad12507-bib-0090] Santilli, S. , S. Grossen , and L. Nota . 2020. “Career Adaptability, Resilience, and Life Satisfaction Among Italian and Belgian Middle School Students.” The Career Development Quarterly 68, no. 3: 194–207.

[jad12507-bib-0091] Santilli, S. , J. Marcionetti , S. Rochat , J. Rossier , and L. Nota . 2017. “Career Adaptability, Hope, Optimism, and Life Satisfaction in Italian and Swiss Adolescents.” Journal of Career Development 44, no. 1: 62–76.

[jad12507-bib-0092] Santilli, S. , L. Nota , M. C. Ginevra , and S. Soresi . 2014. “Career Adaptability, Hope and Life Satisfaction in Workers With Intellectual Disability.” Journal of Vocational Behavior 85: 67–74.

[jad12507-bib-0093] Sapienza, J. K. , and A. S. Masten . 2011. “Understanding and Promoting Resilience in Children and Youth.” Current Opinion in Psychiatry 24, no. 4: 267–273. 21546838 10.1097/YCO.0b013e32834776a8

[jad12507-bib-0094] Savickas, M. L. 2007. “Internationalisation of Counseling Psychology: Constructing Cross‐National Consensus and Collaboration.” Applied Psychology 56, no. 1: 182–188.

[jad12507-bib-0095] Savickas, M. L. 2012. “Life Design: A Paradigm for Career Intervention in the 21st Century.” Journal of Counseling & Development 90, no. 1: 13–19.

[jad12507-bib-0096] Savickas, M. L. , L. Nota , J. Rossier , et al. 2009. “Life Designing: A Paradigm for Career Construction in the 21st Century.” Journal of Vocational Behavior 75, no. 3: 239–250.

[jad12507-bib-0097] Savickas, M. L. , and E. J. Porfeli . 2012. “Career Adapt‐Abilities Scale: Construction, Reliability, and Measurement Equivalence Across 13 Countries.” Journal of Vocational Behavior 80, no. 3: 661–673.

[jad12507-bib-0098] Savickas, M. L. 2013. “Career Construction Theory and Practice.” In Career Development and Counseling: Putting Theory and Research to Work, edited by S. Brown and R. Lent , 147–183. John Wiley & Sons Inc.

[jad12507-bib-0099] Savickas, M. L. 2005. “The Theory and Practice of Career Construction.” In Career Development and Counseling: Putting Theory and Research to Work, edited by S. D. Brown and R. W. Lent , 42–70. John Wiley & Sons, Inc.

[jad12507-bib-0100] Schneider, S. L. 2001. “In Search of Realistic Optimism: Meaning, Knowledge, and Warm Fuzziness.” American Psychologist 56, no. 3: 250–263. 11315251 10.1037//0003-066x.56.3.250

[jad12507-bib-0101] Schultheiss, O. C. 2008. “Implicit Motives.” In Handbook of Personality: Theory and Research, edited by O. P. John , R. W. Robins and L. A. Pervin , 603–633. The Guilford Press.

[jad12507-bib-0102] Schwab, K. 2016. The Fourth Industrial Revolution: What It Means, How to Respond. https://www.weforum.org/agenda/2016/01/the-fourth-industrial-revolution-what-it‐means-and-how-to-respond/.

[jad12507-bib-0103] Schwarz, G. 1978. “Estimating the Dimension of a Model.” Annals of Statistics: 461–464.

[jad12507-bib-0104] Sclove, S. L. 1987. “Application of Model‐Selection Criteria to Some Problems in Multivariate Analysis.” Psychometrika 52: 333–343.

[jad12507-bib-0105] Snyder, C. R. 2002. “TARGET ARTICLE: Hope Theory: Rainbows in the Mind.” Psychological inquiry 13, no. 4: 249–275.

[jad12507-bib-0106] Snyder, C. R. , C. Harris , J. R. Anderson , et al. 1991. “The Will and the Ways: Development and Validation of an Individual‐Differences Measure of Hope.” Journal of Personality and Social Psychology 60, no. 4: 570–585. 2037968 10.1037//0022-3514.60.4.570

[jad12507-bib-0107] Soresi, S. , L. Nota , and L. Ferrari . 2012. “Career Adapt‐Abilities Scale‐Italian Form: Psychometric Properties and Relationships to Breadth of Interests, Quality of Life, and Perceived Barriers.” Journal of Vocational Behavior 80, no. 3: 705–711.

[jad12507-bib-0108] Spurk, D. , A. Hirschi , M. Wang , D. Valero , and S. Kauffeld . 2020. “Latent Profile Analysis: A Review and “How to” Guide of Its Application Within Vocational Behavior Research.” Journal of Vocational Behavior 120: 103445.

[jad12507-bib-0109] Sumer, M. , F. Giannotta , M. Settanni , and S. Ciairano . 2009. “Parental Support as Mediator Between Optimism and Depression in Early Adolescents.” Journal of Psychology and Counseling 1, no. 8: 139–146.

[jad12507-bib-0110] Teixeira, M. A. P. , M. P. Bardagi , M. C. P. Lassance , M. O. Magalhães , and M. E. Duarte . 2012. “Career Adapt‐Abilities Scale‐Brazilian Form: Psychometric Properties and Relationships to Personality.” Journal of Vocational Behavior 80, no. 3: 680–685.

[jad12507-bib-0111] Tolentino, L. R. , V. Sedoglavich , V. N. Lu , P. R. J. M. Garcia , and S. L. D. Restubog . 2014. “The Role of Career Adaptability in Predicting Entrepreneurial Intentions: A Moderated Mediation Model.” Journal of Vocational Behavior 85, no. 3: 403–412.

[jad12507-bib-0113] Urbanaviciute, I. , A. Kairys , B. Pociute , and A. Liniauskaite . 2014. “Career Adaptability in Lithuania: A Test of Psychometric Properties and a Theoretical Model.” Journal of Vocational Behavior 85, no. 3: 433–442.

[jad12507-bib-0114] van Vianen, A. E. M. , U. C. Klehe , J. Koen , and N. Dries . 2012. “Career Adapt‐Abilities Scale—Netherlands Form: Psychometric Properties and Relationships to Ability, Personality, and Regulatory Focus.” Journal of Vocational Behavior 80, no. 3: 716–724.

[jad12507-bib-0115] Wang, M. , and P. J. Hanges . 2011. “Latent Class Procedures: Applications to Organizational Research.” Organizational Research Methods 14, no. 1: 24–31.

[jad12507-bib-0116] Wehmeyer, M. L. , L. Nota , S. Soresi , et al. 2019. “A Crisis In Career Development: Life Designing and Implications for Transition.” Career Development and Transition for Exceptional Individuals 42, no. 3: 179–187.

[jad12507-bib-0117] Wolfe, J. B. , and N. E. Betz . 2004. “The Relationship of Attachment Variables to Career Decision‐Making Self‐Efficacy and Fear of Commitment.” The Career Development Quarterly 52, no. 4: 363–369.

[jad12507-bib-1004] Wong, W. L. L. , and S. H. Cheung . 2024. “The Role of Hope in College Transition: Its Cross‐Lagged Relationships With Psychosocial Resources and Emotional Well‐Being in First‐Year College Students.” Journal of Adolescence 96, no. 4: 771–788. 38287896 10.1002/jad.12297

[jad12507-bib-0118] WorldSkills & Organization for Economic Co‐operation and Development (OECD) . 2019. Youth Voice for the Future of Work 2019. OECD.

[jad12507-bib-0119] Xu, C. , X. Gong , W. Fu , et al. 2020. “The Role of Career Adaptability and Resilience in Mental Health Problems in Chinese Adolescents.” Children and Youth Services Review 112: 104893.

[jad12507-bib-0120] Zammitti, A. , A. Russo , M. C. Ginevra , and P. Magnano . 2023. “‘Imagine Your Career After the COVID‐19 Pandemic': An Online Group Career Counseling Training for University Students.” Behavioral Sciences 13, no. 1: 48. 36661620 10.3390/bs13010048 PMC9855113

[jad12507-bib-0121] Zeng, Q. , J. Li , S. Huang , et al. 2023. “How Does Career‐Related Parental Support Enhance Career Adaptability: The Multiple Mediating Roles of Resilience and Hope.” Current Psychology 42, no. 29: 25193–25205.

[jad12507-bib-0122] Zhou, H. , Q. Li , J. Shen , J. Jia , W. Tong , and X. Fang . 2024. “A Transactional Model of Career Adaptability: Longitudinal Links Between Parental Career‐Related Behaviors, Parent‐Child Relationship, and Career Adaptability Among Chinese Adolescents.” Journal of Vocational Behavior 153: 104025.

